# Chloridotris(penta­fluoro­benzene­thiol­ato-κ*S*)[tris­(4-fluoro­phen­yl)phosphine-κ*P*]osmium(IV)

**DOI:** 10.1107/S1600536810011281

**Published:** 2010-03-31

**Authors:** Asdrúbal Arias, Lidia Meléndez, Sylvain Bernès, Maribel Arroyo

**Affiliations:** a Centro de Química del Instituto de Ciencias, BUAP, Ciudad Universitaria, San Manuel, 72570 Puebla, Pue., Mexico; b DEP Facultad de Ciencias Químicas, UANL, Guerrero y Progreso S/N, Col. Treviño, 64570 Monterrey, N.L., Mexico

## Abstract

The title complex, [Os(C_6_F_5_S)_3_Cl(C_18_H_12_F_3_P)], displays a trigonal-bipyramidal Os^IV^ coordination geometry with the S atoms of three thiol­ate ligands occupying the equatorial positions. The thiol­ate penta­fluoro­phenyl substituents are all placed above the equatorial plane, forming a claw-like cavity which accommodates the chloride ligand with a normal Os—Cl bond length. The phosphine ligand *trans* to the chloride ligand reveals a short Os—P bond length compared to other chloride–phosphine Os^IV^ complexes (average = 2.40 Å). This strong bonding indicates that the inductive effect of the F atoms in the phosphine does not affect significantly its basicity, compared to triphenyl­phosphine. This feature is also consistent with the known poor *trans* influence of Cl^−^. The crystal packing involves π–π contacts between inversion-related thiol­ate C_6_F_5_ rings, with a centroid–centroid separation of 3.659 (8) Å.

## Related literature

For the structures of related five-coordinated Os^IV^ complexes, see: Hills *et al.* (1991 ▶); Arroyo *et al.* (1994 ▶, 2007 ▶, 2009 ▶); Cerón *et al.* (2006 ▶); Mendoza *et al.* (2006 ▶). For the structure and basicity of free tris­(4-fluoro­phen­yl)phosphine, see: bin Shawkataly *et al.* (1996 ▶) and Allman & Goel (1982 ▶), respectively. For geometrical analysis using the Cambridge Structural Database, see: Bruno *et al.* (2002 ▶).
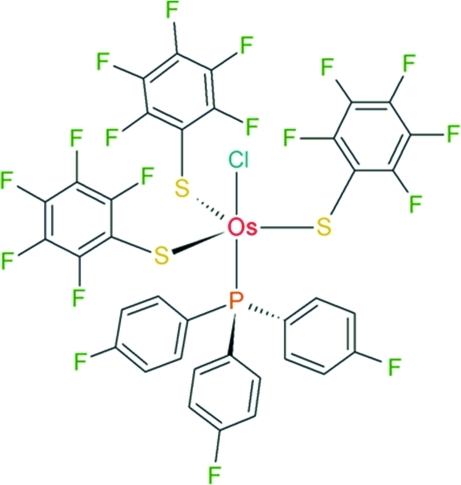


## Experimental

### 

#### Crystal data

[Os(C_6_F_5_S)_3_Cl(C_18_H_12_F_3_P)]
*M* *_r_* = 1139.26
Monoclinic, 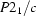

*a* = 17.983 (7) Å
*b* = 10.446 (5) Å
*c* = 21.521 (8) Å
β = 107.62 (2)°
*V* = 3853 (3) Å^3^
*Z* = 4
Mo *K*α radiation
μ = 3.70 mm^−1^
*T* = 298 K
0.5 × 0.4 × 0.3 mm

#### Data collection

Siemens P4 diffractometer
Absorption correction: ψ scan (*XSCANS*; Siemens, 1996 ▶) *T*_min_ = 0.215, *T*_max_ = 0.330
13694 measured reflections
6749 independent reflections
5120 reflections with *I* > 2σ(*I*)
*R*_int_ = 0.052
3 standard reflections every 97 reflections  intensity decay: 0.5%

#### Refinement

*R*[*F* ^2^ > 2σ(*F* ^2^)] = 0.048
*wR*(*F* ^2^) = 0.135
*S* = 1.33
6749 reflections
542 parameters
H-atom parameters constrained
Δρ_max_ = 1.96 e Å^−3^
Δρ_min_ = −2.11 e Å^−3^

### 

Data collection: *XSCANS* (Siemens, 1996 ▶); cell refinement: *XSCANS*; data reduction: *XSCANS*; program(s) used to solve structure: *SHELXTL-Plus* (Sheldrick, 2008 ▶); program(s) used to refine structure: *SHELXTL-Plus*; molecular graphics: *Mercury* (Macrae *et al.*, 2008 ▶); software used to prepare material for publication: *SHELXTL-Plus*.

## Supplementary Material

Crystal structure: contains datablocks I, global. DOI: 10.1107/S1600536810011281/kp2255sup1.cif

Structure factors: contains datablocks I. DOI: 10.1107/S1600536810011281/kp2255Isup2.hkl

Additional supplementary materials:  crystallographic information; 3D view; checkCIF report

## Figures and Tables

**Table 1:** Selected bond lengths (Å)

Os1—S1	2.205 (3)
Os1—S2	2.199 (3)
Os1—S3	2.206 (3)
Os1—Cl1	2.414 (2)
Os1—P1	2.334 (2)

**Fig. 1.**
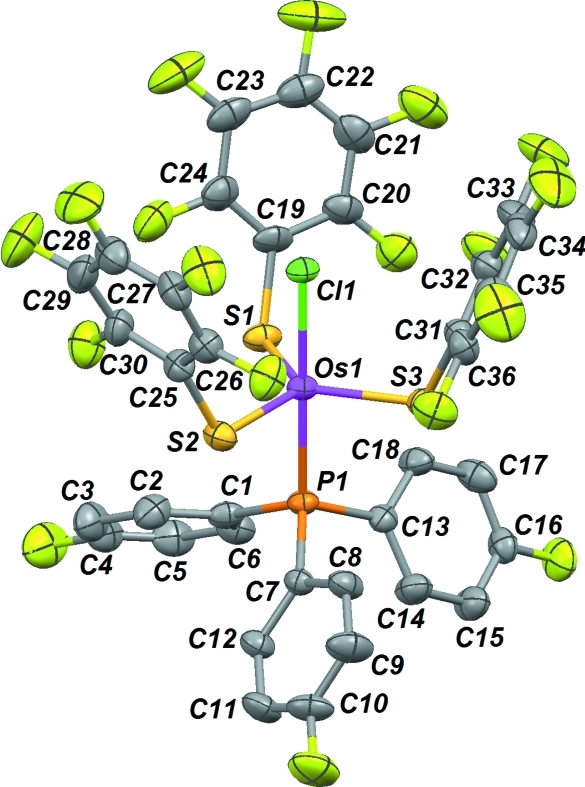
The structure of the title compound with displacement ellipsoids at the 30% probability level for non-H atoms.

**Fig. 2.**
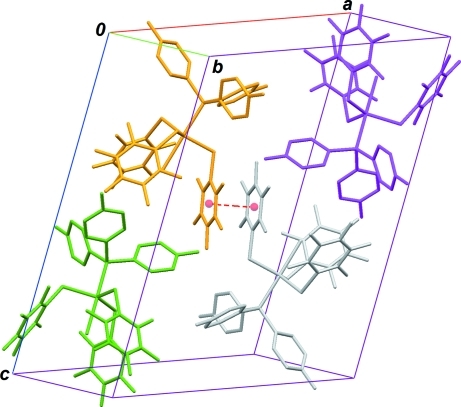
Part of the crystal structure with the shortest π–π contact showed by dashed line. Symmetry related molecules are represented by different colours; grey: asymmetric unit; gold: 1-x, 1-y, 1-z; green: 1-x, -1/2+y, 3/2-z; purple: x, 3/2-y, -1/2+z.

## supplementary crystallographic information

### Comment

We have been interested for a long time in Os^IV^ complexes bearing fluorinated thiolate ligands, as these systems are involved in the study of the C—F bond activation (Arroyo *et al.*, 2007, and references therein). For the five-coordinated complexes with general formula [Os^IV^(thiolate)_4_(phosphine)], the metal centre is invariably found to be in a trigonal-bipyramidal geometry (*e.g.* Mendoza *et al.*, 2006; Cerón *et al.*, 2006; Arroyo *et al.*, 2009), with the phosphine and one thiolate groups placed in axial positions. Of interest are the complexes [Os*X*(thiolate)_3_(phosphine)], where *X* is an halogen *trans* to the phosphine in a bipyramidal geometry (Hills *et al.*, 1991; Arroyo *et al.*, 1994)and halides are known to have little *trans* influence, allowing the estimation of the basicity of the coordinated phosphine.

The title complex was obtained by reaction of [Os(SC_6_F_5_)_4_(P(C_6_H_4_F-4)_3_)] (space group *P*1, Arroyo *et al.*, 2009) and HCl(aq) in acetone (see *Experimental*). The complex approximates 3-fold symmetry (Fig. 1), with atoms P1 and Cl1 placed in axial positions. The coordinated Cl^-^ ion is placed in a cavity approximating point symmetry *C*_3_, formed by the C_6_F_5_ groups of the thiolate ligands, all 'up' towards and around the smaller chloride ligand. The trigonal-bipyramidal coordination geometry of the Os^IV^ centre is completed by three S atoms in the equatorial plane.

The chloride ligand is found at the expected distance from the metal center, Os—Cl = 2.414 (2) Å, which compares well with the average Os—Cl bond length retrieved from 804 hits in the CSD, 2.401 Å (CSD, version 5.31 with all updates; Bruno *et al.*, 2002). A quite different situation is observed for the phosphine: the Os—P bond is short, 2.334 (2) Å, compared to other Os^IV^ complexes including the P—Os—Cl fragment (*ca*. 2.40 Å). For all the 340 hits retrieved in the CSD with a P—Os—Cl fragment, the average Os—P bond length is 2.383 (2) Å. The phosphine is thus strongly bonded to the metal centre in the title compound. In spite of the inductive effect of the F atoms in the phosphine, a significant amount of electron density should be donated back to the rings and the P atom, favoring the ligand bonding. This behaviour is consistent with the p*K*_a_ of that phosphine, 1.97, which is close to the p*K*_a_ of triphenylphosphine, 2.73 (Allman & Goel, 1982). Finally, the strong coordination of tris(4-fluorophenyl)phosphine in the title compound also affects the geometry of this ligand: short P—C bond lengths are observed, in the range 1.787 (10)–1.797 (10) Å, while the mean P—C distance reported in the free tris(4-fluorophenyl)phosphine is 1.825 (5) Å (bin Shawkataly *et al.*, 1996).

The crystal structure is stabilized by intermolecular π–π interactions involving C_6_F_5_ rings of thiolate ligands related by an inversion centre (Fig. 2). The separation between the centroids of stacked rings is 3.659 (8) Å.

### Experimental

The previously prepared complex [Os(SC_6_F_5_)_4_(P(C_6_H_4_F-4)_3_)] (Arroyo *et al.*, 2009) (0.200 g, 0.154 mmol) was dissolved in acetone (50 ml), and HCl 1.5 M (5 ml) was added. The mixture was stirred at room temperature for *ca*. 48 h, monitoring the progress of the reaction by TLC. The solvent was distilled off under vacuum and the solid product was purified through a silica gel chromatographic column eluted with hexane-dichloromethane (4:1), affording [OsCl(SC_6_F_5_)_3_(P(C_6_H_4_F)_3_)] (75% yield). Complex decomposes at 423 K, with colour changing from dark-brown to black, and does not show defined melting point. FAB-MS {m/z (%) [fragment]}: 1139 (2) [M^+^], 1121 (4) [M^+^—F], 1105 (65) [M^+^—Cl], 1045 (4) [M^+^—C_6_H_4_F], 941 (100) [M^+^—SC_6_F_5_], 923 (9) [M^+^—SC_6_F_5_—F], 391 (34) [M^+^—P(C_6_H_4_F)_3_-2SC_6_F_5_—Cl]. Single crystals were obtained by slow evaporation of the eluent at room temperature.

### Refinement

Diffraction data were collected at room temperature, and completeness was reduced to 0.99 because 68 reflections were rejected during data reduction. As commonly found in perfluorinated complexes, F atoms display high displacement parameters. As a consequence, significant Hirshfeld differences are observed for some C—C and C—F bonds in the thiolate groups. Limited accuracy is also reflected in the small C—C average bond length in benzene ring C31···C36 (*ca*. 1.36 Å). H atoms were placed in idealized positions, with C—H bond lengths fixed to 0.93 Å, and *U*_iso_(H) = 1.2 *U*_eq_(carrier C).

### Crystal data

**Table:**

[Os(C_6_F_5_S)_3_Cl(C_18_H_12_F_3_P)]	*F*(000) = 2184
*M**_r_* = 1139.26	*D*_x_ = 1.964 Mg m^−^^3^
Monoclinic, *P*2_1_/*c*	Mo *K*α radiation, λ = 0.71073 Å
Hall symbol: -P 2ybc	Cell parameters from 84 reflections
*a* = 17.983 (7) Å	θ = 5.2–12.5°
*b* = 10.446 (5) Å	µ = 3.70 mm^−^^1^
*c* = 21.521 (8) Å	*T* = 298 K
β = 107.62 (2)°	Prism, dark-brown
*V* = 3853 (3) Å^3^	0.5 × 0.4 × 0.3 mm
*Z* = 4	

### Data collection

**Table:**

Siemens P4 diffractometer	5120 reflections with *I* > 2σ(*I*)
Radiation source: fine-focus sealed tube	*R*_int_ = 0.052
graphite	θ_max_ = 25.0°, θ_min_ = 2.0°
ω scans	*h* = −21→11
Absorption correction: ψ scan (*XSCANS*; Siemens, 1996)	*k* = −12→1
*T*_min_ = 0.215, *T*_max_ = 0.330	*l* = −24→25
13694 measured reflections	3 standard reflections every 97 reflections
6749 independent reflections	intensity decay: 0.5%

### Refinement

**Table:**

Refinement on *F*^2^	Secondary atom site location: difference Fourier map
Least-squares matrix: full	Hydrogen site location: inferred from neighbouring sites
*R*[*F*^2^ > 2σ(*F*^2^)] = 0.048	H-atom parameters constrained
*wR*(*F*^2^) = 0.135	*w* = 1/[σ^2^(*F*_o_^2^) + (0.026*P*)^2^ + 24.9505*P*] where *P* = (*F*_o_^2^ + 2*F*_c_^2^)/3
*S* = 1.33	(Δ/σ)_max_ = 0.002
6749 reflections	Δρ_max_ = 1.96 e Å^−^^3^
542 parameters	Δρ_min_ = −2.11 e Å^−^^3^
0 restraints	Extinction correction: *SHELXTL-Plus* (Sheldrick, 2008), Fc^*^=kFc[1+0.001xFc^2^λ^3^/sin(2θ)]^-1/4^
0 constraints	Extinction coefficient: 0.00165 (11)
Primary atom site location: structure-invariant direct methods	

### Fractional atomic coordinates and isotropic or equivalent isotropic displacement parameters (Å^2^)

**Table:**

	*x*	*y*	*z*	*U*_iso_*/*U*_eq_	
Os1	0.76039 (2)	0.56074 (3)	0.715615 (17)	0.04286 (15)	
Cl1	0.78380 (14)	0.6117 (2)	0.61374 (11)	0.0508 (6)	
P1	0.73741 (14)	0.5076 (2)	0.81355 (11)	0.0428 (5)	
S1	0.78307 (18)	0.7581 (3)	0.75283 (13)	0.0589 (7)	
S2	0.85683 (16)	0.4221 (3)	0.74230 (12)	0.0572 (6)	
S3	0.63988 (15)	0.4992 (3)	0.66494 (12)	0.0549 (6)	
C1	0.8010 (6)	0.5869 (9)	0.8837 (5)	0.053 (2)	
C2	0.8829 (7)	0.5691 (12)	0.8998 (5)	0.072 (3)	
H2A	0.9022	0.5170	0.8732	0.086*	
C3	0.9347 (8)	0.6259 (14)	0.9531 (6)	0.082 (4)	
H3A	0.9883	0.6133	0.9632	0.099*	
C4	0.9038 (9)	0.7011 (13)	0.9902 (5)	0.076 (4)	
F4	0.9545 (6)	0.7575 (9)	1.0432 (4)	0.120 (3)	
C5	0.8274 (9)	0.7246 (12)	0.9780 (5)	0.076 (4)	
H5A	0.8099	0.7783	1.0051	0.092*	
C6	0.7743 (7)	0.6666 (11)	0.9236 (5)	0.066 (3)	
H6A	0.7211	0.6819	0.9144	0.079*	
C7	0.7474 (5)	0.3408 (9)	0.8337 (4)	0.045 (2)	
C8	0.7133 (7)	0.2499 (10)	0.7861 (5)	0.063 (3)	
H8A	0.6900	0.2766	0.7434	0.075*	
C9	0.7135 (9)	0.1221 (12)	0.8007 (6)	0.083 (4)	
H9A	0.6925	0.0622	0.7682	0.099*	
C10	0.7446 (9)	0.0850 (11)	0.8631 (6)	0.075 (4)	
F10	0.7452 (6)	−0.0411 (7)	0.8774 (4)	0.110 (3)	
C11	0.7818 (7)	0.1675 (11)	0.9110 (6)	0.073 (3)	
H11A	0.8066	0.1385	0.9530	0.087*	
C12	0.7819 (6)	0.2956 (11)	0.8956 (5)	0.057 (3)	
H12A	0.8062	0.3534	0.9283	0.069*	
C13	0.6421 (5)	0.5476 (10)	0.8168 (4)	0.048 (2)	
C14	0.6033 (7)	0.4718 (12)	0.8503 (6)	0.071 (3)	
H14A	0.6261	0.3952	0.8685	0.085*	
C15	0.5324 (7)	0.5062 (13)	0.8573 (6)	0.075 (3)	
H15A	0.5088	0.4560	0.8817	0.090*	
C16	0.4974 (6)	0.6154 (12)	0.8278 (5)	0.060 (3)	
F16	0.4288 (4)	0.6488 (8)	0.8350 (4)	0.089 (2)	
C17	0.5316 (7)	0.6903 (12)	0.7939 (6)	0.074 (3)	
H17A	0.5066	0.7644	0.7742	0.089*	
C18	0.6040 (6)	0.6581 (11)	0.7881 (5)	0.061 (3)	
H18A	0.6273	0.7111	0.7647	0.073*	
C19	0.7813 (7)	0.8609 (9)	0.6869 (5)	0.062 (3)	
C20	0.7086 (8)	0.9076 (11)	0.6483 (6)	0.070 (3)	
F20	0.6449 (5)	0.8800 (8)	0.6635 (4)	0.092 (2)	
C21	0.7051 (9)	0.9799 (12)	0.5942 (7)	0.084 (4)	
F21	0.6339 (6)	1.0171 (9)	0.5564 (4)	0.120 (3)	
C22	0.7686 (11)	1.0100 (13)	0.5801 (7)	0.088 (4)	
F22	0.7646 (7)	1.0821 (8)	0.5264 (4)	0.137 (4)	
C23	0.8428 (10)	0.9711 (13)	0.6153 (7)	0.083 (4)	
F23	0.9054 (6)	0.9989 (10)	0.6012 (5)	0.133 (4)	
C24	0.8443 (8)	0.8931 (12)	0.6702 (6)	0.076 (3)	
F24	0.9150 (5)	0.8441 (9)	0.7039 (4)	0.100 (3)	
C25	0.9160 (6)	0.4406 (10)	0.6912 (5)	0.054 (2)	
C26	0.9050 (6)	0.3713 (11)	0.6354 (5)	0.059 (3)	
F26	0.8450 (4)	0.2905 (7)	0.6162 (4)	0.088 (2)	
C27	0.9505 (7)	0.3856 (13)	0.5952 (5)	0.070 (3)	
F27	0.9364 (5)	0.3208 (10)	0.5399 (4)	0.107 (3)	
C28	1.0114 (7)	0.4685 (15)	0.6120 (6)	0.078 (4)	
F28	1.0582 (5)	0.4826 (11)	0.5748 (5)	0.125 (3)	
C29	1.0258 (7)	0.5396 (14)	0.6685 (8)	0.085 (4)	
F29	1.0851 (5)	0.6206 (11)	0.6855 (5)	0.138 (4)	
C30	0.9755 (6)	0.5254 (12)	0.7056 (6)	0.067 (3)	
F30	0.9929 (4)	0.5951 (8)	0.7607 (4)	0.095 (2)	
C31	0.6220 (6)	0.4927 (10)	0.5789 (5)	0.054 (2)	
C32	0.5926 (7)	0.5966 (12)	0.5394 (6)	0.069 (3)	
F32	0.5806 (5)	0.7067 (7)	0.5659 (4)	0.104 (3)	
C33	0.5783 (8)	0.5886 (16)	0.4740 (6)	0.085 (4)	
F33	0.5500 (6)	0.6898 (11)	0.4365 (4)	0.139 (4)	
C34	0.5880 (7)	0.4766 (19)	0.4461 (6)	0.087 (4)	
F34	0.5699 (6)	0.4657 (13)	0.3809 (4)	0.140 (4)	
C35	0.6150 (8)	0.3715 (15)	0.4836 (6)	0.081 (4)	
F35	0.6243 (6)	0.2614 (11)	0.4560 (4)	0.133 (4)	
C36	0.6326 (6)	0.3817 (11)	0.5496 (5)	0.063 (3)	
F36	0.6620 (5)	0.2803 (7)	0.5865 (4)	0.088 (2)	

### Atomic displacement parameters (Å^2^)

**Table:**

	*U*^11^	*U*^22^	*U*^33^	*U*^12^	*U*^13^	*U*^23^
Os1	0.0543 (2)	0.0379 (2)	0.0388 (2)	−0.00019 (17)	0.01766 (16)	0.00087 (15)
Cl1	0.0629 (14)	0.0505 (13)	0.0431 (12)	−0.0031 (11)	0.0224 (11)	0.0043 (10)
P1	0.0553 (14)	0.0410 (13)	0.0371 (12)	−0.0003 (11)	0.0212 (10)	0.0003 (10)
S1	0.0863 (19)	0.0437 (14)	0.0519 (15)	−0.0079 (13)	0.0288 (14)	−0.0035 (11)
S2	0.0636 (15)	0.0599 (17)	0.0513 (14)	0.0123 (13)	0.0223 (12)	0.0082 (12)
S3	0.0617 (15)	0.0576 (16)	0.0481 (14)	−0.0056 (12)	0.0207 (12)	0.0031 (11)
C1	0.078 (7)	0.036 (5)	0.050 (6)	−0.007 (5)	0.026 (5)	−0.004 (4)
C2	0.085 (8)	0.078 (8)	0.058 (7)	−0.017 (7)	0.030 (6)	−0.016 (6)
C3	0.087 (9)	0.093 (10)	0.058 (7)	−0.027 (8)	0.008 (6)	−0.018 (7)
C4	0.099 (10)	0.079 (9)	0.043 (6)	−0.036 (8)	0.008 (6)	−0.013 (6)
F4	0.151 (8)	0.130 (8)	0.067 (5)	−0.062 (6)	0.017 (5)	−0.037 (5)
C5	0.116 (11)	0.064 (8)	0.051 (7)	−0.015 (7)	0.027 (7)	−0.022 (6)
C6	0.087 (8)	0.055 (7)	0.056 (6)	0.003 (6)	0.023 (6)	−0.002 (5)
C7	0.050 (5)	0.043 (5)	0.043 (5)	0.005 (4)	0.016 (4)	0.002 (4)
C8	0.083 (8)	0.051 (6)	0.045 (6)	−0.007 (6)	0.004 (5)	0.004 (5)
C9	0.134 (12)	0.043 (6)	0.070 (8)	−0.006 (7)	0.031 (8)	0.001 (6)
C10	0.123 (11)	0.041 (6)	0.071 (8)	0.014 (6)	0.045 (7)	0.015 (6)
F10	0.157 (8)	0.052 (5)	0.108 (6)	0.005 (5)	0.020 (5)	0.025 (4)
C11	0.095 (9)	0.057 (7)	0.057 (7)	0.013 (6)	0.009 (6)	0.019 (6)
C12	0.070 (7)	0.057 (7)	0.041 (5)	0.004 (5)	0.011 (5)	0.005 (5)
C13	0.053 (5)	0.056 (6)	0.035 (5)	−0.004 (5)	0.014 (4)	−0.004 (4)
C14	0.073 (7)	0.060 (7)	0.087 (8)	0.005 (6)	0.035 (7)	0.018 (6)
C15	0.061 (7)	0.078 (8)	0.096 (9)	0.014 (6)	0.039 (7)	0.020 (7)
C16	0.048 (6)	0.075 (8)	0.057 (6)	0.012 (5)	0.017 (5)	−0.004 (6)
F16	0.058 (4)	0.112 (6)	0.103 (5)	0.023 (4)	0.034 (4)	0.006 (5)
C17	0.090 (9)	0.072 (8)	0.064 (7)	0.028 (7)	0.028 (6)	0.010 (6)
C18	0.066 (7)	0.062 (7)	0.066 (7)	0.007 (5)	0.034 (5)	0.015 (5)
C19	0.101 (9)	0.030 (5)	0.055 (6)	−0.023 (5)	0.023 (6)	−0.007 (4)
C20	0.101 (10)	0.041 (6)	0.064 (7)	−0.006 (6)	0.019 (7)	−0.001 (5)
F20	0.094 (5)	0.073 (5)	0.112 (6)	0.012 (4)	0.034 (5)	0.008 (4)
C21	0.101 (11)	0.049 (7)	0.088 (10)	0.010 (7)	0.009 (8)	−0.003 (6)
F21	0.152 (8)	0.083 (6)	0.105 (6)	0.023 (6)	0.008 (6)	0.017 (5)
C22	0.130 (13)	0.060 (8)	0.088 (10)	−0.022 (9)	0.057 (10)	−0.014 (7)
F22	0.274 (13)	0.072 (6)	0.078 (5)	−0.008 (7)	0.074 (7)	0.013 (4)
C23	0.116 (12)	0.058 (8)	0.092 (10)	−0.036 (8)	0.054 (9)	−0.021 (7)
F23	0.179 (9)	0.118 (8)	0.142 (8)	−0.070 (7)	0.108 (7)	−0.026 (6)
C24	0.089 (9)	0.062 (8)	0.074 (8)	−0.030 (7)	0.020 (7)	−0.011 (6)
F24	0.091 (5)	0.106 (7)	0.110 (6)	−0.040 (5)	0.038 (5)	−0.015 (5)
C25	0.052 (5)	0.063 (7)	0.054 (6)	0.014 (5)	0.027 (5)	−0.002 (5)
C26	0.064 (6)	0.056 (7)	0.058 (6)	0.007 (5)	0.020 (5)	−0.007 (5)
F26	0.083 (5)	0.086 (5)	0.098 (5)	−0.016 (4)	0.033 (4)	−0.034 (4)
C27	0.063 (7)	0.090 (9)	0.053 (6)	0.022 (7)	0.010 (5)	−0.008 (6)
F27	0.103 (6)	0.149 (8)	0.076 (5)	0.023 (5)	0.036 (4)	−0.028 (5)
C28	0.067 (8)	0.100 (11)	0.073 (8)	0.020 (7)	0.028 (7)	0.007 (7)
F28	0.114 (7)	0.165 (9)	0.131 (7)	0.015 (6)	0.091 (6)	0.022 (7)
C29	0.054 (7)	0.081 (10)	0.116 (11)	−0.007 (7)	0.018 (7)	0.013 (8)
F29	0.095 (6)	0.150 (9)	0.180 (10)	−0.057 (6)	0.059 (6)	−0.036 (8)
C30	0.053 (6)	0.075 (8)	0.069 (7)	−0.014 (6)	0.011 (5)	−0.010 (6)
F30	0.087 (5)	0.103 (6)	0.089 (5)	−0.017 (4)	0.016 (4)	−0.033 (4)
C31	0.057 (6)	0.054 (6)	0.049 (6)	0.000 (5)	0.011 (5)	0.003 (5)
C32	0.072 (7)	0.068 (8)	0.065 (7)	0.004 (6)	0.015 (6)	0.007 (6)
F32	0.143 (7)	0.061 (5)	0.092 (5)	0.021 (5)	0.009 (5)	0.008 (4)
C33	0.086 (9)	0.099 (11)	0.061 (8)	0.003 (8)	0.005 (7)	0.030 (8)
F33	0.158 (9)	0.145 (9)	0.093 (6)	0.021 (7)	0.007 (6)	0.065 (6)
C34	0.064 (7)	0.144 (15)	0.047 (7)	−0.012 (9)	0.009 (6)	0.004 (8)
F34	0.134 (8)	0.236 (13)	0.047 (4)	0.001 (8)	0.022 (4)	−0.007 (6)
C35	0.089 (9)	0.096 (11)	0.064 (8)	0.003 (8)	0.030 (7)	−0.019 (8)
F35	0.151 (8)	0.149 (9)	0.099 (6)	0.022 (7)	0.038 (6)	−0.058 (6)
C36	0.061 (6)	0.060 (7)	0.061 (7)	0.007 (5)	0.010 (5)	−0.004 (6)
F36	0.112 (6)	0.059 (4)	0.096 (5)	0.018 (4)	0.034 (4)	−0.002 (4)

### Geometric parameters (Å, °)

**Table:**

Os1—S1	2.205 (3)	C16—F16	1.336 (11)
Os1—S2	2.199 (3)	C16—C17	1.339 (16)
Os1—S3	2.206 (3)	C17—C18	1.386 (15)
Os1—Cl1	2.414 (2)	C17—H17A	0.9300
Os1—P1	2.334 (2)	C18—H18A	0.9300
P1—C13	1.787 (10)	C19—C24	1.331 (16)
P1—C7	1.791 (10)	C19—C20	1.407 (17)
P1—C1	1.797 (10)	C20—F20	1.316 (14)
S1—C19	1.772 (10)	C20—C21	1.374 (18)
S2—C25	1.759 (9)	C21—C22	1.306 (19)
S3—C31	1.783 (10)	C21—F21	1.349 (15)
C1—C6	1.382 (14)	C22—F22	1.362 (16)
C1—C2	1.420 (15)	C22—C23	1.38 (2)
C2—C3	1.375 (16)	C23—F23	1.284 (15)
C2—H2A	0.9300	C23—C24	1.429 (18)
C3—C4	1.354 (18)	C24—F24	1.360 (15)
C3—H3A	0.9300	C25—C30	1.350 (15)
C4—C5	1.340 (18)	C25—C26	1.364 (14)
C4—F4	1.360 (12)	C26—F26	1.333 (12)
C5—C6	1.405 (16)	C26—C27	1.366 (15)
C5—H5A	0.9300	C27—F27	1.326 (13)
C6—H6A	0.9300	C27—C28	1.356 (18)
C7—C12	1.372 (13)	C28—F28	1.334 (13)
C7—C8	1.395 (14)	C28—C29	1.381 (19)
C8—C9	1.371 (15)	C29—F29	1.323 (15)
C8—H8A	0.9300	C29—C30	1.384 (17)
C9—C10	1.345 (16)	C30—F30	1.346 (13)
C9—H9A	0.9300	C31—C36	1.360 (15)
C10—F10	1.352 (13)	C31—C32	1.381 (15)
C10—C11	1.354 (17)	C32—F32	1.329 (14)
C11—C12	1.379 (15)	C32—C33	1.355 (17)
C11—H11A	0.9300	C33—F33	1.334 (15)
C12—H12A	0.9300	C33—C34	1.35 (2)
C13—C18	1.388 (14)	C34—F34	1.346 (14)
C13—C14	1.391 (14)	C34—C35	1.36 (2)
C14—C15	1.376 (15)	C35—F35	1.328 (16)
C14—H14A	0.9300	C35—C36	1.364 (16)
C15—C16	1.363 (16)	C36—F36	1.333 (13)
C15—H15A	0.9300		
			
S2—Os1—S1	118.47 (11)	F16—C16—C17	120.2 (11)
S2—Os1—S3	121.08 (11)	F16—C16—C15	118.5 (10)
S1—Os1—S3	119.87 (11)	C17—C16—C15	121.3 (10)
S2—Os1—P1	87.06 (9)	C16—C17—C18	120.3 (11)
S1—Os1—P1	87.61 (9)	C16—C17—H17A	119.8
S3—Os1—P1	87.77 (9)	C18—C17—H17A	119.8
S2—Os1—Cl1	92.40 (9)	C17—C18—C13	120.9 (10)
S1—Os1—Cl1	93.42 (9)	C17—C18—H18A	119.6
S3—Os1—Cl1	91.75 (9)	C13—C18—H18A	119.6
P1—Os1—Cl1	178.96 (9)	C24—C19—C20	117.9 (11)
C13—P1—C7	104.2 (5)	C24—C19—S1	124.0 (11)
C13—P1—C1	103.5 (5)	C20—C19—S1	118.1 (9)
C7—P1—C1	104.7 (5)	F20—C20—C21	120.8 (13)
C13—P1—Os1	114.5 (3)	F20—C20—C19	120.1 (10)
C7—P1—Os1	114.7 (3)	C21—C20—C19	119.1 (13)
C1—P1—Os1	114.1 (3)	C22—C21—F21	121.9 (15)
C19—S1—Os1	108.6 (3)	C22—C21—C20	120.7 (15)
C25—S2—Os1	110.1 (4)	F21—C21—C20	117.4 (14)
C31—S3—Os1	111.5 (4)	C21—C22—F22	120.2 (17)
C6—C1—C2	117.2 (10)	C21—C22—C23	124.7 (14)
C6—C1—P1	123.2 (9)	F22—C22—C23	115.0 (14)
C2—C1—P1	119.6 (8)	F23—C23—C22	125.1 (14)
C3—C2—C1	122.5 (12)	F23—C23—C24	121.7 (16)
C3—C2—H2A	118.7	C22—C23—C24	113.2 (13)
C1—C2—H2A	118.7	C19—C24—F24	119.7 (12)
C4—C3—C2	116.5 (13)	C19—C24—C23	124.3 (14)
C4—C3—H3A	121.7	F24—C24—C23	115.9 (13)
C2—C3—H3A	121.7	C30—C25—C26	116.5 (10)
C5—C4—C3	124.7 (11)	C30—C25—S2	121.0 (8)
C5—C4—F4	118.2 (12)	C26—C25—S2	122.5 (9)
C3—C4—F4	117.1 (13)	F26—C26—C25	119.8 (10)
C4—C5—C6	118.9 (11)	F26—C26—C27	117.3 (10)
C4—C5—H5A	120.6	C25—C26—C27	122.8 (11)
C6—C5—H5A	120.6	F27—C27—C28	119.0 (12)
C1—C6—C5	120.1 (12)	F27—C27—C26	121.5 (12)
C1—C6—H6A	120.0	C28—C27—C26	119.5 (11)
C5—C6—H6A	120.0	F28—C28—C27	121.1 (13)
C12—C7—C8	116.6 (9)	F28—C28—C29	119.0 (14)
C12—C7—P1	123.5 (8)	C27—C28—C29	119.9 (11)
C8—C7—P1	119.7 (7)	F29—C29—C28	120.6 (13)
C9—C8—C7	121.6 (10)	F29—C29—C30	121.5 (14)
C9—C8—H8A	119.2	C28—C29—C30	118.0 (12)
C7—C8—H8A	119.2	F30—C30—C25	121.0 (10)
C10—C9—C8	118.7 (12)	F30—C30—C29	115.6 (11)
C10—C9—H9A	120.6	C25—C30—C29	123.2 (12)
C8—C9—H9A	120.6	C36—C31—C32	117.8 (10)
C9—C10—F10	118.7 (12)	C36—C31—S3	120.5 (8)
C9—C10—C11	122.4 (11)	C32—C31—S3	121.6 (9)
F10—C10—C11	118.6 (11)	F32—C32—C33	119.6 (12)
C10—C11—C12	118.1 (10)	F32—C32—C31	119.9 (10)
C10—C11—H11A	121.0	C33—C32—C31	120.5 (12)
C12—C11—H11A	121.0	F33—C33—C34	119.7 (13)
C7—C12—C11	122.3 (10)	F33—C33—C32	119.9 (15)
C7—C12—H12A	118.9	C34—C33—C32	120.4 (13)
C11—C12—H12A	118.9	F34—C34—C33	120.9 (15)
C18—C13—C14	116.5 (9)	F34—C34—C35	118.6 (16)
C18—C13—P1	121.8 (7)	C33—C34—C35	120.4 (12)
C14—C13—P1	121.7 (8)	F35—C35—C34	120.3 (13)
C15—C14—C13	122.3 (11)	F35—C35—C36	120.8 (14)
C15—C14—H14A	118.8	C34—C35—C36	118.9 (13)
C13—C14—H14A	118.8	F36—C36—C31	119.2 (10)
C16—C15—C14	118.7 (11)	F36—C36—C35	118.9 (11)
C16—C15—H15A	120.7	C31—C36—C35	121.9 (12)
C14—C15—H15A	120.7		
			
S2—Os1—P1—C13	−160.3 (4)	S1—C19—C20—C21	−175.4 (9)
S1—Os1—P1—C13	81.0 (4)	F20—C20—C21—C22	178.3 (12)
S3—Os1—P1—C13	−39.0 (4)	C19—C20—C21—C22	−3.0 (19)
S2—Os1—P1—C7	−39.9 (4)	F20—C20—C21—F21	−2.3 (18)
S1—Os1—P1—C7	−158.6 (4)	C19—C20—C21—F21	176.4 (10)
S3—Os1—P1—C7	81.4 (4)	F21—C21—C22—F22	1(2)
S2—Os1—P1—C1	80.8 (4)	C20—C21—C22—F22	−179.7 (12)
S1—Os1—P1—C1	−37.9 (4)	F21—C21—C22—C23	−177.5 (13)
S3—Os1—P1—C1	−157.9 (4)	C20—C21—C22—C23	2(2)
S2—Os1—S1—C19	109.5 (5)	C21—C22—C23—F23	179.0 (13)
S3—Os1—S1—C19	−79.1 (5)	F22—C22—C23—F23	1(2)
P1—Os1—S1—C19	−165.2 (5)	C21—C22—C23—C24	0(2)
Cl1—Os1—S1—C19	14.9 (5)	F22—C22—C23—C24	−178.5 (11)
S1—Os1—S2—C25	−81.7 (4)	C20—C19—C24—F24	−176.3 (10)
S3—Os1—S2—C25	107.0 (4)	S1—C19—C24—F24	1.3 (16)
P1—Os1—S2—C25	−167.3 (4)	C20—C19—C24—C23	−0.6 (18)
Cl1—Os1—S2—C25	13.5 (4)	S1—C19—C24—C23	177.0 (9)
S2—Os1—S3—C31	−85.5 (4)	F23—C23—C24—C19	−179.7 (11)
S1—Os1—S3—C31	103.3 (4)	C22—C23—C24—C19	−0.5 (19)
P1—Os1—S3—C31	−170.7 (4)	F23—C23—C24—F24	−3.8 (18)
Cl1—Os1—S3—C31	8.4 (4)	C22—C23—C24—F24	175.3 (11)
C13—P1—C1—C6	−4.8 (10)	Os1—S2—C25—C30	87.0 (10)
C7—P1—C1—C6	−113.7 (9)	Os1—S2—C25—C26	−93.0 (9)
Os1—P1—C1—C6	120.2 (8)	C30—C25—C26—F26	−176.5 (10)
C13—P1—C1—C2	175.6 (9)	S2—C25—C26—F26	3.5 (15)
C7—P1—C1—C2	66.7 (9)	C30—C25—C26—C27	−0.3 (17)
Os1—P1—C1—C2	−59.4 (9)	S2—C25—C26—C27	179.7 (9)
C6—C1—C2—C3	1.1 (18)	F26—C26—C27—F27	−0.8 (17)
P1—C1—C2—C3	−179.3 (10)	C25—C26—C27—F27	−177.1 (11)
C1—C2—C3—C4	0(2)	F26—C26—C27—C28	178.8 (11)
C2—C3—C4—C5	−1(2)	C25—C26—C27—C28	2.5 (19)
C2—C3—C4—F4	179.8 (12)	F27—C27—C28—F28	−2.0 (19)
C3—C4—C5—C6	1(2)	C26—C27—C28—F28	178.3 (11)
F4—C4—C5—C6	−179.7 (11)	F27—C27—C28—C29	178.2 (12)
C2—C1—C6—C5	−1.0 (16)	C26—C27—C28—C29	−1(2)
P1—C1—C6—C5	179.4 (9)	F28—C28—C29—F29	0(2)
C4—C5—C6—C1	0.1 (18)	C27—C28—C29—F29	179.9 (13)
C13—P1—C7—C12	−94.5 (9)	F28—C28—C29—C30	178.6 (12)
C1—P1—C7—C12	13.8 (10)	C27—C28—C29—C30	−2(2)
Os1—P1—C7—C12	139.6 (8)	C26—C25—C30—F30	−177.6 (10)
C13—P1—C7—C8	80.0 (9)	S2—C25—C30—F30	2.4 (16)
C1—P1—C7—C8	−171.7 (8)	C26—C25—C30—C29	−3.0 (18)
Os1—P1—C7—C8	−46.0 (9)	S2—C25—C30—C29	177.0 (10)
C12—C7—C8—C9	0.8 (17)	F29—C29—C30—F30	−3(2)
P1—C7—C8—C9	−174.0 (10)	C28—C29—C30—F30	178.9 (12)
C7—C8—C9—C10	3(2)	F29—C29—C30—C25	−177.6 (12)
C8—C9—C10—F10	−179.6 (12)	C28—C29—C30—C25	4(2)
C8—C9—C10—C11	−6(2)	Os1—S3—C31—C36	92.6 (9)
C9—C10—C11—C12	5(2)	Os1—S3—C31—C32	−91.3 (9)
F10—C10—C11—C12	179.0 (11)	C36—C31—C32—F32	179.4 (11)
C8—C7—C12—C11	−1.4 (16)	S3—C31—C32—F32	3.3 (16)
P1—C7—C12—C11	173.2 (9)	C36—C31—C32—C33	−2.8 (18)
C10—C11—C12—C7	−1.5 (19)	S3—C31—C32—C33	−178.9 (10)
C7—P1—C13—C18	−162.8 (8)	F32—C32—C33—F33	−2(2)
C1—P1—C13—C18	87.9 (9)	C31—C32—C33—F33	−179.9 (12)
Os1—P1—C13—C18	−36.8 (9)	F32—C32—C33—C34	−178.1 (13)
C7—P1—C13—C14	19.5 (10)	C31—C32—C33—C34	4(2)
C1—P1—C13—C14	−89.8 (10)	F33—C33—C34—F34	0(2)
Os1—P1—C13—C14	145.5 (8)	C32—C33—C34—F34	176.3 (12)
C18—C13—C14—C15	−2.7 (18)	F33—C33—C34—C35	−178.4 (13)
P1—C13—C14—C15	175.1 (10)	C32—C33—C34—C35	−2(2)
C13—C14—C15—C16	3(2)	F34—C34—C35—F35	1(2)
C14—C15—C16—F16	−179.7 (11)	C33—C34—C35—F35	179.8 (13)
C14—C15—C16—C17	−1.7 (19)	F34—C34—C35—C36	−179.2 (11)
F16—C16—C17—C18	177.9 (10)	C33—C34—C35—C36	0(2)
C15—C16—C17—C18	0.0 (19)	C32—C31—C36—F36	178.9 (10)
C16—C17—C18—C13	0.4 (18)	S3—C31—C36—F36	−4.9 (15)
C14—C13—C18—C17	0.9 (16)	C32—C31—C36—C35	−0.1 (18)
P1—C13—C18—C17	−176.9 (9)	S3—C31—C36—C35	176.1 (10)
Os1—S1—C19—C24	−92.9 (10)	F35—C35—C36—F36	2.4 (19)
Os1—S1—C19—C20	84.7 (9)	C34—C35—C36—F36	−177.3 (11)
C24—C19—C20—F20	−178.9 (11)	F35—C35—C36—C31	−178.5 (12)
S1—C19—C20—F20	3.3 (14)	C34—C35—C36—C31	2(2)
C24—C19—C20—C21	2.3 (16)		
